# Psychometric Properties of the Schizophrenia Oral Health Profile: Preliminary Results

**DOI:** 10.3390/ijerph18179090

**Published:** 2021-08-28

**Authors:** Frédéric Denis, Ines Rouached, Francesca Siu-Paredes, Alexis Delpierre, Gilles Amador, Wissam El-Hage, Nathalie Rude

**Affiliations:** 1 Faculty of Dentistry, Nantes University, 44000 Nantes, France; gilles.amadordelvalle@chu-nantes.fr; 2 Department of Odontology, Tours University Hospital, 37000 Tours, France; alexis.delpierre@outlook.fr; 3 EA 75-05 Education, Ethique, Santé, Faculté de Médecine, Université de Tours, 37000 Tours, France; 4 EA 481 Integrative and Clinical Neurosciences, University of Besançon, 25000 Besançon, France; ines.rouached@edu.univ-fcomte.fr (I.R.); chinitasiu@hotmail.com (F.S.-P.); nathalie.retel-rude@univ-fcomte.fr (N.R.); 5 Faculté d’Odontologie de Reims, Université Champagne Ardenne, 51100 Reims, France; 6 U1253, iBrain, CIC1415, Inserm, CHRU de Tours (Regional University Hospital Centre), Université de Tours, 37000 Tours, France; wissam.elhage@univ-tours.fr

**Keywords:** schizophrenia, psychometric, oral health-related quality of life, oral health

## Abstract

The Schizophrenia Oral Health Profile questionnaire was developed to assess the oral health-related quality of life among individuals with schizophrenia based on their perceptions rather than from caregivers. A 5-point Likert scale was used to self-report on 42 items. In the present study, different analyses were conducted to determine the dimensional structure of the final scale: (1) inter-item correlation analysis and Cronbach’s α coefficient, (2) Rasch model analysis, (3) exploratory factor analysis and (4) confirmatory factor analysis. The final version of the Schizophrenia Oral Health Profile questionnaire consisted of 20 items and an internal structure composed of three dimensions: (1) emotions related to oral health, (2) oral pain and discomfort and (3) self-image, others’ views and the need for care. We showed that the difficulty and discrimination indices of each of the 20 selected items were acceptable according to the Rasch model, as well as their inter-item and inter-score correlations (α = 0.875). The psychometric study of the Schizophrenia Oral Health Profile questionnaire is still in progress to investigate reproducibility, sensitivity to change and external structure.

## 1. Introduction

When people suffer from a severe mental illness such as schizophrenia, it is difficult to know how they perceive their physical symptoms and, more broadly, how they feel in terms of their health needs and quality of life. Indeed, positive symptoms such as hallucinations and dysfunctional thought patterns, negative symptoms associated with emotional and behavioural disturbances and cognitive symptoms manifest as difficulties in comprehension, decision-making and attention, which combine to deleteriously impact the daily functioning of these people [1]. In this context, people with schizophrenia (PWS) may have difficulties in expressing their physical health needs and accessing health services. It is estimated that, in this population, half of the physical disorders (e.g., cardiovascular, gastrointestinal, respiratory, neoplastic, infectious, endocrine and oral) are undiagnosed [2,3].

The oral health of PWS is poor compared with that of the general population, and indices of dental caries and periodontal measurements are often twice the level found in the general population [4,5,6]. We now know that many factors combine to contribute to poor oral health in these individuals. These factors include poor diet and lifestyle behaviours (high-sugar diet, use of psychoactive substances such as tobacco and inadequate oral hygiene) as well as the side effects of antipsychotic treatments [7]. It is also worth mentioning that the weight of stigmatisation or self-stigmatisation limits patient access to the health care system [3,8].

Knowing the perceived oral health needs as well as the representations and perceptions related to oral disorders or oral health-related quality of life (OHrQoL) in schizophrenia-related disorders is essential to effective care and to build adapted care strategies but also to develop prevention and promotion programmes for this health issue. To evaluate the relevance of these actions, reliable and valid measurement tools are needed. Even though we have established the reliability and validity of the Global Oral Health Assessment Index (GOHAI) scale in a French representative sample of PWS [9], the side effects of psychostimulants or antipsychotics, which include trismus, facial muscle pain, myasthenia or dyskinesia (tremors) or drooling with clozapine, are not captured by this scale.

A recent study presents the different stages of the development of the “Schizophrenia Oral Health Profile” (SOHP) questionnaire, which aims to assess oral disorders and their impact on the psychosocial functioning and well-being of PWS [10]. This work was conducted in close collaboration with health professionals and PWS. The approach in establishing the SOHP questionnaire was based on comments made by patients (74% of the items were generated by PWS) and health professionals during semi-structured interviews. The results of this study led to the construction of a provisional self-questionnaire composed of 42 items assessed using a 5-point Likert scale from which three assumed dimensions emerged: one dimension related to pain, one related to oral dysfunctions and the last related to the psychosocial impacts of oral disease.

The present study aimed to investigate the psychometric properties of the provisional SOHP questionnaire.

## 2. Materials and Methods

### 2.1. Research Design

As a first step, from June 2016 to November 2018, a qualitative study was conducted to explore the importance values of OHrQOL for PWS. From this study, a conceptual questionnaire of 42 items was obtained. This first step was conducted at the Chartreuse Hospital (Dijon, France) with 34 people (24 PWS and 10 health professionals) [10]. In a second step, the psychometric properties (internal consistency and reliability) of this questionnaire were explored with a large sample of SPWs recruited in five French hospitals from September 2018 to June 2021. The psychometric study of the SOHP scale is ongoing to investigate the reproducibility, sensitivity to change and external structure of this scale. The study was registered with www.ClinicalTrials.gov, accessed on 9 October 2018 under the number NCT03699501.

**Figure 1:**
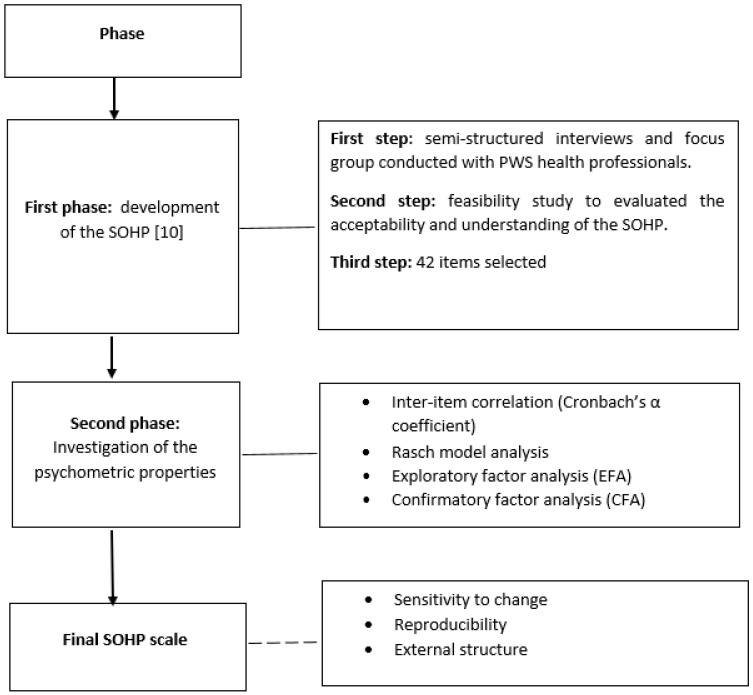
Process of program development and psychometric testing. SOHP: Schizophrenia Oral Health Profile; PWS: people with schizophrenia.

Below, Figure 1 summarises the research design used in the present study.

### 2.2. Analysis

To investigate the psychometric properties of the provisional SOHP questionnaire, we used (1) inter-item correlation analysis and Cronbach’s α coefficient, (2) Rasch model analysis, (3) exploratory factor analysis (EFA) and (4) confirmatory factor analysis (CFA).

#### 2.2.1. Inter-Item Correlation and Cronbach’s α Coefficient

Internal consistency was assessed using Cronbach’s α coefficient and inter-item correlation [11,12]. Cronbach’s α values greater than 0.75 indicate excellent reliability, values between 0.40 and 0.75 show fair to good reliability, and values less than 0.40 indicate poor reliability [11,12].

#### 2.2.2. Rasch Model Analysis

The Rasch model was used to examine how well the provisional SOHP questionnaire functioned as a measurement of oral disorders and their impact on the psychosocial functioning and well-being of PWS by analysing how closely the observed item ratings matched those expected by the model. The Rasch model considered the different aspects of the measurement system, including items and rating scale categories, influencing the score [13]. As the aim was to refine the provisional instrument, the analysis was primarily used to highlight items or rating categories that showed substantial ‘misfit’ to the model, suggesting they may not usefully contribute, or may even degrade, the instrument’s performance as a measurement system, and may need deleting.

#### 2.2.3. Exploratory Factor Analysis (EFA)

EFA is a common technique used to explore the characteristics of an instrument and guide its development without any assumptions about the number or structure of this instrument [14,15]. EFA is often used in addition to Rasch analysis [16]. This analysis also aims to determine whether the correlations between items justify the belief that the items measure the same trait. If the items are not unidimensional, the scale may need to be divided into subscales, or items may need to be deleted (Kaiser-Meyer-Olkin-MO, value > 0.6 and Bartlett test results showing significant sphericity) [14,15,16].

#### 2.2.4. Confirmatory Factor Analysis (CFA)

CFA was conducted to check the data collected and the stability of the factor structure of the final version of the SOHP scale. The goodness of fit was assessed using chi-square/df, root mean square error of approximation (RMSEA), comparative fit index (CFI) and IFI (incremental fit index) [17].

### 2.3. Sample

The evaluation of the SOHP scale was conducted with PWS in several psychiatric institutions (Dijon, Tours, Millau, Reims, Paris). The expected number of subjects using G * Power R (wp.logistic) [15], a sample size program based on Cohen’s sampling formula, was set at a significance level of 0.05, a power of 0.80 and an effect size of 0.30 per 100 persons. The PWS were selected between September 2018 and June 2021.

### 2.4. Ethical Considerations

This study, named “Quality bis”, was approved by the Committee for the Protection of Persons of the Ile de France region (registration number: 2018-A02043-52). After providing participants with a complete description of the study, informed consent was obtained from each participant or their legal guardians for individuals under guardianship. In the latter case, the patient’s legal guardian(s) signed the informed consent.

### 2.5. Data Analysis

The analysis was conducted using software package R (Bell Laboratories, New Providence, NJ, USA).

## 3. Results

### 3.1. Participants’ General Characteristics

For the 102 PWS who participated in the “Quality bis” study, there was very little missing data from the 42-item SOHP questionnaire (less than 5%), see Table 1.

### 3.2. The Psychometric Properties of the Provisional SOHP Questionnaire (42 Items)

#### 3.2.1. Inter-Item Correlation

The correlation of each item (1 to 42) of the SOHP is presented in Table 2.

Inter-item correlations are an essential element in conducting an item analysis of a set of test questions. Inter-item correlations examine the extent to which scores on one item are related to scores on all other items on a scale. Ideally, the average inter-item correlation for a set of items should be between 0.20 and 0.40, suggesting that while the items are reasonably homogenous, they do contain sufficiently unique variance so as to not be isomorphic with each other. When values are lower than 0.20, then the items may not be representative of the same content domain. If values are higher than 0.40, the items may be only capturing a small bandwidth of the construct.

#### 3.2.2. Rasch Model Analysis

The average of the values of the response modalities was between 1 and 4, with standard deviations varying between 0.5 and 1.5 for each item (Table 3).

Initially, the items with a discrimination index that was negative or close to 0 were removed (S3 and S42). Then, the misfit items that did not meet the standard criteria of fit statistics were excluded [13]. Thus, items S2, S3, S4, S5, S6, S7, S9, S13, S23, S28, S29, S30, S31, S32, S33, S34, S35, S36, S38, S39, S40 and S42 were excluded from the scale. The SOHP was then reduced from 42 to 20 items.

#### 3.2.3. Exploratory Factor Analysis (EFA)

**Figure 2:**
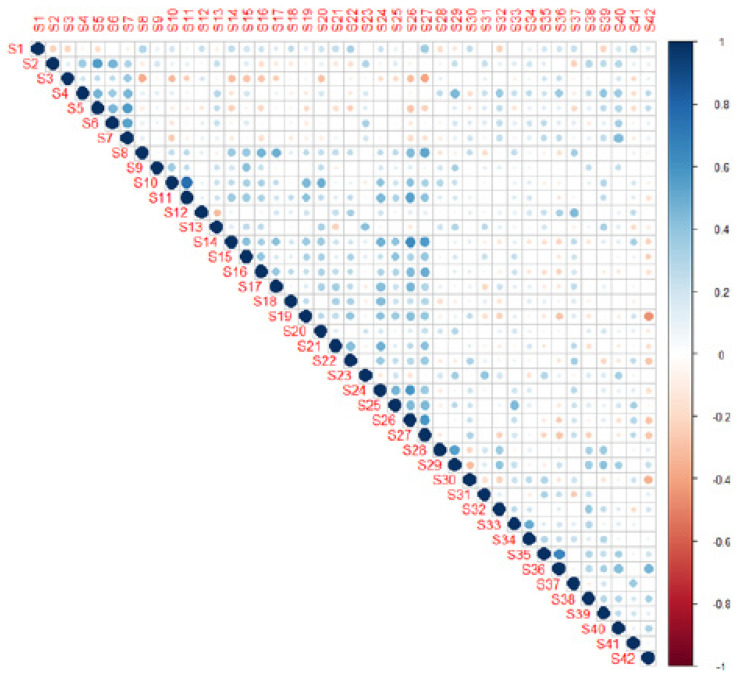
The SOHP item contribution matrix.

EFA was performed on the 42 items to search for possible dimensions and to confirm the exclusion of the 22 items previously removed after the Rasch analysis. Figure 2 allows us to determine by which factor (or factorial dimension) the items are best represented.

**Figure 3:**
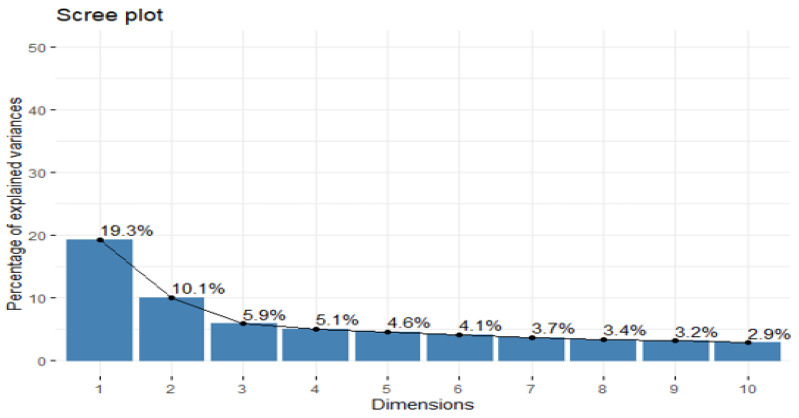
SOHP (42 items) eigenvalues graph.

Figure 3 shows that the first axis of the PCA concentrates 19.3% of the information. The second, third, fourth, fifth and sixth axes each contain 10.10%, 5.9%, 5.1%, 4.6% and 4.1%, respectively. We have about 49.1% of the total variance explained by the first six axes.

#### 3.2.4. Confirmatory Factor Analysis (CFA)

**Figure 4:**
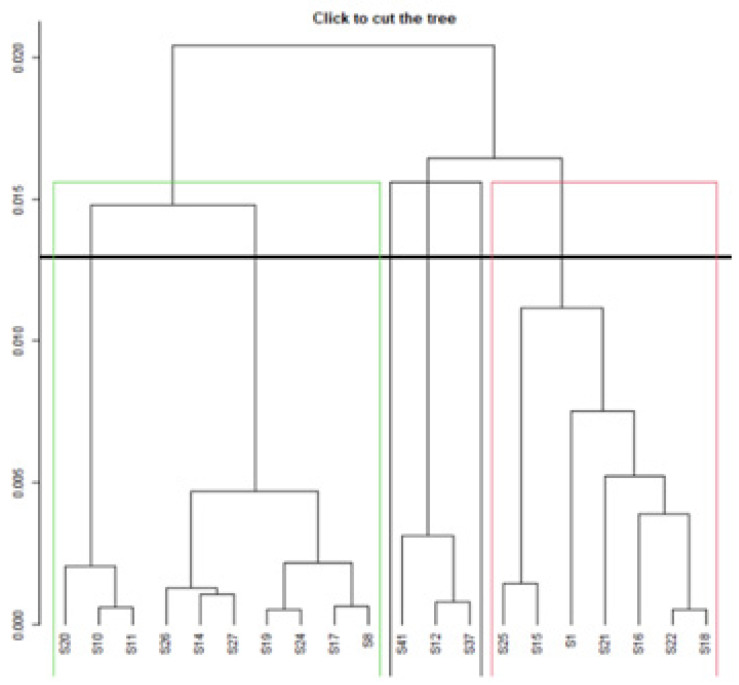
Individual items dendrogram for the SOHP scale with 20 items.

We found that the Rasch model eliminated 20 items outside the initial concept of quality of life. CFA was conducted to elicit more precise results on the validity of the 20-item SOHP. In the hierarchical analysis, we found that three groups were formed (Figure 4).

Figure 4 shows that items S8, 10, 11, 14, 17, 19, 20, 24, 26 and 27 were grouped in cluster 1. Items S12, 37 and 41 were grouped in cluster 2 and items S1, 15, 16, 18, 21, 22 and 25 were grouped in cluster 3.

### 3.3. The Psychometric Properties of the Final SOHP Questionnaire (20 Items)

#### 3.3.1. Inter-Item Correlation

The correlation of each item (1 to 20) the SOHP score is presented in Table 4.

#### 3.3.2. Rasch Model Analysis

The results of the Rasch model analysis are presented in Table 5.

#### 3.3.3. The Three Dimensions of the Final Version of the SOHP (20 Items)

As the factor analysis did not generate sub-themes (dimensions), we hypothesised that the items were grouped according to the proximity of their conceptual content. Therefore, we chose to construct dimensions based on conceptual similarity. Three dimensions emerged more precisely than in the first version of the questionnaire: (1) emotions related to oral health, (2) oral pain and discomfort and (3) self-image, others’ views and the need for care.

Dimension 1 included seven items, S1, S12, S19, S20, S25, S26 and S37, all associated with the concept of emotions related to oral health (Table 6).

Dimension 2 included eight items, S8, S10, S16, S17, S18, S21, S22 and S24, all associated with the concept of oral pain and discomfort (Table 7).

Dimension 3 included five items, S11, S14, S15, S27 and S41, all associated with the concept of self-image, the gaze of others and the need for care (Table 8).

## 4. Discussion

The SOHP was developed for measuring the OHrQoL of PWS. We showed that the difficulty and discrimination indices of each of the 20 items selected for the final version are acceptable according to the Rasch model, as are the inter-item (0.258) and inter-score correlations (α = 0.875). We highlighted three dimensions in the final version of the psychometrically validated SOHP, (1) emotions related to oral health, (2) oral pain and discomfort and (3) self-image, others’ view and the need for care.

Regarding dimension 1, the effects of oral health conditions on physical and psychosocial dimensions have been a topic of interest for several authors over the last decades [20,21]. Like Settineri et al. [22], we found a significant relationship between the patient’s perception of oral health and their mood states: “Item 25: I am worried about my oral health”, “Item 26. I am embarrassed to speak because of the condition of my mouth”, “Item 19: I have bad breath”. Item 20: “I grind my teeth, highlights a physical expression of stress and/or anxiety in the form of teeth grinding” [23]. These items allow a better understanding of the psychological mechanisms involved in treatment adherence and should allow the carers to focus on the elements of the oral disorder that are most meaningful to the patient rather than what they think is best.

Teeth have a symbolic value in emotional life insofar as the mouth, the main organ of our expressive capacity, can be read scientifically as an organ to be treated or symbolically as a part of the body capable of recording and expressing our psycho-emotional experience [21,22]. This is documented in “Item 27: I am embarrassed to speak because of the state of my mouth” and “Item 25: I am worried about my oral health” by PWS. In this context, attention to the role of emotions in oral life should involve both psychologists and dentists to maintain the psychosocial, physical and emotional well-being of PWS regarding oral health care.

More generally, emotional state is evidenced by PWS in the form of anxiety attacks (Item 1: I have anxiety attacks) and mood disorders (Item 12: I am lost in thought, replaying memories). Our results suggest that the pattern of stress experienced by PWS may be generated in part by poor oral health and that further research could contribute to psychosocial interventions to improve oral health (Item 37: I need help to manage my feelings). Oral health is severely impaired in these populations [4,5,6,7]. The SOHP scale will help to encourage caregivers to develop interpersonal skills (regular dental visits, brushing twice a day, reducing sugar intake…) to help restore oral health/global health.

The notion of pain and discomfort is a topic clearly expressed by PWS (Item 8: “My teeth hurt”; Item 16 “My mouth is sensitive to hot and cold”) as in the general population. These data are interesting because it has long been thought that behavioural pain reactivity and self-reported pain responses are reduced in schizophrenia even though there is little or no physiological evidence to support pain insensitivity in PWS. The reduced sensitivity to pain in schizophrenia is thought to be related more to a different mode of pain expression than to actual endogenous analgesia [24]. To our knowledge, this is the first time that facial pain has been documented by an OHrQoL questionnaire (Item 10: “I have facial muscle pain”). This type of pain can be explained by the fact that these patients are exposed to a wide variety of drugs that may contribute to the increased occurrence of orofacial pain such as temporomandibular disorders [25].

In addition to pain, people with PWS also express their feelings about the oral cavity in detail (Item 17: “It hurts when I brush my teeth”). These complaints often take a back seat in PWS, in which stabilisation of symptoms remains the priority. Functional disorders (Item 22: “My mouth is dry or sticky”; Item 24: “I have difficulty chewing”) highlight very specific disorders related to the use of anticholinergic drugs, which lead to a decrease in salivary secretion or even an alteration in salivary composition that is very disabling in daily life [26]. Saliva is a natural lubricant in the oral cavity and aids in the early stages of digestion. Surprisingly, among the discomforts felt, PWS express discomfort related to bleeding gums (Item 21: “My gums bleed”), which shows the attention that these people pay to their oral health and calls into question the idea that PWS are detached from somatic disorders [27].

The notion of self-image, the gaze of others, is an essential notion for PWS, as they suffer from stigmatisation and many prejudices [28,29,30]. It is interesting to note that PWS feel that “the gaze of others” clearly passes through the mouth with all the symbolism associated with it in relationships (Item 11: “My jaw and my teeth are not good”; Item14: “Because of the appearance of my mouth, I am afraid of the gaze of others”; Item 27: “The state of my mouth makes it difficult for me to smile”). PWS expressed the need for support in managing their oral health (Item 15: “I need care for my oral well-being (for my oral health)”; Item 41: “I need help to manage my health”). Mental illness greatly impacts daily life management, and seeking help is a first step on the road to the recovery of oral/mental/global health.

## 5. Limitations

This study has some limitations related to the participants’ subjectivity towards oral disorders and their attention span and reasoning ability, given the stage of their mental illness. In addition, some patients were recruited during the COVID period, which exacerbated anxiety and depressive disorders and rendered dental practices difficult to access. It cannot be excluded that the responses to this questionnaire would have been different outside the pandemic. This suggests that follow-up studies with additional CFA should be conducted.

## 6. Conclusions

The SOHP was developed to assess the OHrQoL of PWS. The final version of the SOHP questionnaire consisted of 20 items and an internal structure composed of three dimensions: (1) emotions related to oral health, (2) pain and oral discomfort and (3) self-image, others’ views and the need for care. We showed that the difficulty and discrimination indices of each of the 20 selected items are acceptable according to the Rasch model, as well as their inter-item and inter-score correlations (α = 0.875). The psychometric study of the SOHP scale is still in progress to investigate reproducibility, sensitivity to change and external structure. The SOHP will allow for fine-tuned assessment of the oral health needs of PWS based on their perceptions rather than the point of view of caregivers.

## Figures and Tables

**Table 1:** Participants’ characteristics (*n* = 102).

Variables	Mean ± SD or *n* (%)
Gender (male)	69 (67.6%)
La Chartreuse Hospital Center (male)	8 (61.5%)
Reims University Hospital Center (male)	7 (87.5%)
Tours University Hospital Center (male)	12 (55%)
Millau Hospital Center (male)	26 (78.7)
University Hospital Group Paris Psychiatry & Neurosciences (male)	16 (70%)
Age (years)	40.7 ± 11.5
Marital status	
Married/cohabitation	4 (3.9%)
Single	76 (74.5%)
Duration of psychiatric care (month)	156.3 ± 121.4
Number of antipsychotic treatments	2.0 ± 1.3
Other drug therapy	3.7 ± 3.0
Smoker	46 (45.1%)
Number of cigarettes a day	14.6 ± 8.0
Number of carious teeth	3.1 ± 3.3
Number of missing teeth	5.3 ± 5.0
Number of filled teeth	4.0 ± 5.5
DMFT index *	13.3 ± 7.4
OHI-S index **	1.6 ± 1.0

* DMFT, Decayed, Missing, or Filled Teeth (DMFT) index [18]; ** OHI-S, Simplified Oral Hygiene Index (OHI-S) [19].

**Table 2:** Inter-item correlation for the SOHP (42 items).

SOPH Item	1 0.221	2 0.253	3 −0.022	4 0.306	5 0.088	6 0.204	7 0.128	8 0.436	9 0.248
SOPH Item	10 0.395	11 0.437	12 0.279	13 0.234	14 0.485	15 0.424	16 0.400	17 0.383	18 0.200
SOPH Item	19 0.245	20 0.376	21 0.253	22 0.283	23 0.211	24 0.429	25 0.499	26 0.484	27 0.374
SOPH Item	28 0.239	29 0.389	30 0.279	31 0.122	32 0.199	33 0.440	34 0.391	35 0.212	36 0.238
SOPH Item	37 0.228	38 0.328	39 0.348	40 0.391	41 0.284	42 −0.067			

SOHP: Schizophrenia Oral Health Profile.

**Table 3:** Rasch analysis of the SOHP (42 items).

Item	Missing	Mean	SD	Skew	Kurtosis	W (*p*)	Item Difficulty	Item Discrimination	*α* If Deleted
1: I have anxiety attacks	1.96%	2.2	1.31	0.64	−0.87	0.81 (0.000)	0.44	0.23	0.81
2: I take things easy, without feeling stressed	2.94%	3.39	1.35	−0.5	−0.82	0.87 (0.000)	0.68	0.25	0.81
3: My living conditions are pleasant (food, accommodation, transport)	3.92%	3.49	1.26	−0.68	−0.55	0.86 (0.000)	0.70	−0.12	0.82
4: I engage in physical activity	3.92%	3.55	1.15	−071	−0.10	0.87 (0.000)	0.71	0.41	0.81
5: I feel physically well	3.92%	3.36	1.21	−0.3	−0.80	0.90 (0.000)	0.67	0.09	0.82
6: I sleep well	2.94%	3.86	1.12	−0.84	−0.00	0.85 (0.000)	0.77	0.30	0.81
7: I have a good appetite	1.96%	4	1.12	−1.01	−0.30	0.81 (0.000)	0.80	0.22	0.81
8: My teeth hurt	1.96%	1.99	1.13	1.04	−0.43	0.80 (0.000)	0.40	0.36	0.81
9: My jaw hurts	7.84%	2.93	1.49	0.05	−1.45	0.87 (0.000)	0.59	0.29	0.81
10: I have muscular pain in my face	1.96%	1.45	0.9	2.17	4.39	0.57 (0.000)	0.29	0.40	0.81
11: My jaw and my teeth don’t feel right	2.94%	1.67	1.25	1.71	1.54	0.58 (0.000)	0.33	0.44	0.81
12: I’m lost in thought; I’m rehashing my memories	2.94%	3	1.21	−021	−0.80	0.90 (0.000)	0.60	0.28	0.81
13: I am self-confident, I have good self-esteem	1.96%	3.09	1.16	−0.06	−0.85	0.92 (0.000)	0.62	0.25	0.81
14: Because of how my mouth looks, I’m afraid of how others see me	2.94%	2.12	1.33	0.75	−0.82	0.78 (0.000)	0.42	0.38	0.81
15: I need care for my oral well-being (for my oral and dental health)	1.96%	3.28	1.36	−0.3	−1.10	0.89 (0.000)	0.66	0.44	0.81
16: My mouth is sensitive to hot and cold	2.94%	2.37	1.37	0.54	−0.93	0.84 (0.000)	0.47	0.31	0.81
17: It hurts when I brush my teeth	1.96%	1.7	0.95	1.36	1.59	0.74 (0.000)	0.34	0.34	0.81
18: I find it hard to swallow comfortably	2.94%	1.65	0.93	1.31	1.02	0.71 (0.000)	0.33	0.20	0.81
19: I have bad breath	1.96%	2.06	1.18	0.86	−0.13	0.81 (0.000)	0.41	0.25	0.81
20: I grind my teeth	1.96%	1.59	0.96	1.53	1.41	0.66 (0.000)	0.32	0.37	0.81
21: My gums bleed	1.96%	1.98	1.15	0.86	−0.28	0.80 (0.000)	0.52	0.26	0.81
22: My mouth feels dry or sticky	1.96%	2.6	1.24	−1.21	−1.21	0.87 (0.000)	0.76	0.26	0.81
23: I can articulate sounds correctly	2.94%	3.79	1.29	−0.65	−078	0.83 (0.000)	0.76	0.26	0.81
24: I have difficulty chewing	2.94%	1.93	1.19	1.03	−0.02	0.77 (0.000)	0.39	0.48	0.81
25: I’m worried about my oral health	3.92%	3.05	1.41	−0.11	−1.32	0.88 (0.000)	0.61	0.48	0.81
26: I am embarrassed to speak because of the state of my mouth	2.94%	1.82	1.23	1.36	0.69	0.70 (0.000)	0.36	0.38	0.81
27: I’m embarrassed to smile because of the state of my mouth	3.92%	2.33	1.54	0.61	−1.20	0.77(0.000)	0.47	0.35	0.81
28: I pay attention to my breath	1.96%	3.08	1.45	−0.16	−1.30	0.88 (0.000)	0.62	0.15	0.82
29: I am motivated to brush my teeth	2.94%	3.45	1.43	−0.57	−1.03	0.84 (0.000)	0.69	0.41	0.81
30: I hate brushing my teeth	2.94%	1.51	1	2.06	3.52	0.57 (0.000)	0.30	0.15	0.81
31: I am open to meeting other people	3.92%	3.57	1.24	−0.64	−0.46	0.87 (0.000)	0.71	0.16	0.82
32: I take care of myself (I do my hair, wear make-up, shave…)	2.94%	3.78	1.19	−0.74	−0.40	0.85 (0.000)	0.76	0.21	0.81
33: I talk to my friend(s) about my oral health problems	2.94%	2	1.24	0.86	−0.62	0.77 (0.000)	0.40	0.37	0.81
34: I talk to my family about my oral health problems	22.55%	1.91	1.31	1.21	0.18	0.71 (0.000)	0.38	0.29	0.81
35: I feel that my friend(s) are supportive towards me	2.94%	3.01	1.37	−0.11	−1.14	0.89 (0.000)	0.60	0.23	0.81
36: I feel that my family is supportive towards me	4.90%	3.36	1.49	−0.51	−1.17	0.84 (0.000)	0.67	0.19	0.81
37: I need help to manage my feelings	3.92%	2.5	1.29	0.27	−1.04	0.87 (0.000)	0.50	0.28	0.81
38: I take the time to get the care I need	2.94%	3.58	1.19	−0.69	−0.30	0.87 (0.000)	0.72	0.28	0.81
39: I have projects (for holidays, for work, for my family life)	2.94%	3.08	1.33	−0.23	−1.00	0.89 (0.000)	0.62	0.38	0.81
40: I manage to organise all my activities and appointments	3.92%	3.48	1.33	−0.67	−0.63	0.86 (0.000)	0.70	0.36	0.81
41: I need help to manage my health	2.94%	3.18	1.39	−0.31	−1.10	0.88 (0.000)	0.64	0.23	0.81
42: I’m satisfied with my oral health	4.90%	3.14	1.34	−0.24	−1.09	0.89 (0.000)	0.63	0.00	0.82

SD, standard deviation; mean inter-item correlation = 0.097; Cronbach’s α = 0.816.

**Table 4:** Inter-item correlation for the SOHP 20 items.

SOPH Item	1 0.43	8 0.64	10 0.60	11 0.58	12 0.26	14 0.70	15 0.55	16 0.59	17 0.58	18 0.30
SOPH Item	19 0.56	20 0.33	21 0.40	22 0.57	24 0.66	25 0.43	26 0.75	27 0.70	37 0.42	41 0.40

**Table 5:** Rasch analysis of the 20-item SOHP.

Item	Missing	Mean	SD	Skew	Kurtosis	W (*p*)	Item Difficulty	Item Discrimination	α If Deleted
1	1.96%	2.2	1.31	0.64	−0.87	0.81 (0.000)	0.44	0.40	0.87
8	1.96%	1.99	1.13	1.04	0.43	0.80 (0.000)	0.40	0.58	0.87
10	1.96%	1.45	0.9	2.17	4.39	0.57 (0.000)	0.29	0.51	0.87
11	2.94%	1.67	1.25	1.71	1.54	0.58 (0.000)	0.33	0.48	0.87
12	2.94%	3	1.21	−0.21	−0.80	0.90 (0.000)	0.60	0.28	0.88
14	2.94%	2.12	1.33	0.75	−0.82	0.78 (0.000)	0.42	0.63	0.86
15	1.96%	3.28	1.36	−0.3	−1.10	0.89 (0.000)	0.66	0.54	0.87
16	2.94%	2.37	1.37	−0.54	−0.93	0.84 (0.000)	0.47	0.57	0.86
17	1.96%	1.7	0.95	1.36	1.59	0.74 (0.000)	0.34	0.50	0.87
18	2.94%	1.65	0.93	1.31	1.02	0.71 (0.000)	0.33	0.27	0.88
19	1.96%	2.06	1.18	0.86	−0.13	0.81 (0.000)	0.41	0.50	0.87
20	1.96%	1.59	0.96	1.53	1.41	0.66 (0.000)	0.32	0.23	0.88
21	1.96%	1.98	1.15	0.86	−0.28	0.80 (0.000)	0.40	0.37	0.87
22	1.96%	2.6	1.24	−0.01	−1.21	0.87 (0.000)	0.52	0.54	0.87
24	2.94%	1.93	1.19	1.03	−0.02	0.77 (0.000)	0.39	0.60	0.86
25	3.92%	3.05	1.41	−0.11	−1.32	0.88 (0.000)	0.61	0.41	0.87
26	2.94%	1.82	1.23	1.36	0.69	0.70 (0.000)	0.36	0.67	0.86
27	3.92%	2.33	1.54	0.61	−1.20	0.77 (0.000)	0.47	0.68	0.86
37	3.92%	2.5	1.29	0.27	−1.04	0.87 (0.000)	0.50	0.40	0.87
41	2.94%	3.18	1.39	−0.31	−1.10	0.88 (0.000)	0.64	0.33	0.88

SD, standard deviation; mean inter-item correlation = 0.258; Cronbach’s α = 0.875.

**Table 6:** The concept of emotions related to oral health.

Item	Missing	Mean	SD	Skew	Kurtosis	W (*p*)	Item Difficulty	Item Discrimination	α If Deleted
1	1.96%	2.2	1.31	0.64	−0.87	0.81 (0.000)	0.44	0.23	0.57
12	2.94%	3	1.21	−0.21	−0.80	0.90 (0.000)	0.60	0.30	0.54
19	1.96%	2.06	1.18	0.86	−0.13	0.81 (0.000)	0.41	0.38	0.51
20	1.96%	1.59	0.96	1.53	1.41	0.66 (0.000)	0.32	0.16	0.58
25	3.92%	3.05	1.41	−0.11	−1.32	0.88 (0.000)	0.61	0.30	0.54
26	2.94%	1.82	1.23	1.36	0.69	0.70 (0.000)	0.36	0.40	0.50
37	3.92%	2.5	1.29	0.27	−1.04	0.87 (0.000)	0.50	0.31	0.54

SD, standard deviation; mean inter-item correlation = 0.163; Cronbach’s α = 0.579.

**Table 7:** The concept of oral pain and discomfort.

Item	Missing	Mean	SD	Skew	Kurtosis	W (*p*)	Item Difficulty	Item Discrimination	α If Deleted
8	1.96%	1.99	1.13	1.04	0.43	0.80 (0.000)	0.40	0.56	0.73
10	1.96%	1.45	0.9	2.17	4.39	0.57 (0.000)	0.29	0.43	0.76
16	2.94%	2.37	1.37	0.54	−0.93	0.84 (0.000)	0.47	0.55	0.73
17	1.96%	1.7	0.95	1.36	1.59	0.74 (0.000)	0.34	0.58	0.73
18	2.94%	1.65	0.93	1.31	1.02	0.71 (0.000)	0.33	0.29	0.78
21	1.96%	1.98	1.15	0.86	−0.28	0.80 (0.000)	0.40	0.40	0.76
22	1.96%	2.6	1.24	−0.01	−1.21	0.87 (0.000)	0.52	0.43	0.76
24	2.94%	1.93	1.19	1.03	−0.02	0.77 (0.000)	0.39	0.57	0.73

SD, standard deviation; mean inter-item correlation = 0.298; Cronbach’s α = 0.772.

**Table 8:** The concept of self-image, the gaze of others and the need for care.

Item	Missing	Mean	SD	Skew	Kurtosis	W (*p*)	Item Difficulty	Item Discrimination	α If Deleted
11	2.94%	1.67	1.25	1.71	1.54	0.58 (0.000)	0.33	0.40	0.69
14	2.94%	2.12	1.33	0.75	−0.82	0.78 (0.000)	0.42	0.66	0.58
15	1.96%	3.28	1.36	−0.3	−1.10	0.89 (0.000)	0.66	0.42	0.68
27	3.92%	2.33	1.54	0.61	−1.20	0.77 (0.000)	0.47	0.62	0.59
41	2.94%	3.18	1.39	−031	−1.10	0.88 (0.000)	0.64	0.28	0.74

SD, standard deviation; mean inter-item correlation = 0.328; Cronbach’s α = 0.711.

## Data Availability

The data that support the findings of this study are available from the corresponding author upon reasonable request.
